# Protocol for In Utero Fetal-to-Fetal Kidney Transplantation in Rats

**DOI:** 10.21769/BioProtoc.5565

**Published:** 2026-01-20

**Authors:** Keita Morimoto, Shuichiro Yamanaka, Takashi Yokoo

**Affiliations:** 1 Division of Nephrology and Hypertension, Department of Internal Medicine, The Jikei University School of Medicine, Tokyo, Japan; 2 Kidney Applied Regenerative Medicine, Project Research Units, The Jikei University School of Medicine, Tokyo, Japan

**Keywords:** Fetal kidney transplantation, In utero surgery, Metanephros, Xenotransplantation, Rat model, Potter sequence

## Abstract

Congenital renal disorders, such as the Potter sequence, result from renal dysgenesis. To explore a prenatal therapeutic approach for fetuses with kidney insufficiency, we established an in utero transplantation protocol using donor fetal kidneys. Although numerous rodent studies have reported cellular injections into fetal recipients, no protocol to date has described whole-organ transplantation during gestation. Here, we present a step-by-step method for grafting donor fetal kidneys (embryonic day 14.0–16.5) into allogeneic rat fetuses at embryonic day 18.0–18.5, resulting in term neonates that retain the grafts postnatally. A 15–16 G needle preloaded with the donor kidney is inserted transuterinely, depositing the organ into the subcutaneous space of the fetus. Four days later, the term pups are delivered naturally and evaluated for graft development. This protocol enables organ-level transplantation and longitudinal assessment of graft maturation within the unique fetal environment, which differs markedly from adult settings in terms of growth factor availability and immune reactivity. To our knowledge, this is the first protocol to successfully achieve whole-organ transplantation directly into fetuses in utero. Therefore, the model provides a valuable platform for studying developmental organogenesis, fetal immunology, and regenerative strategies that leverage embryonic cues.

Key features

• Subcutaneous transplantation of fetal kidneys into recipient fetuses minimizes surgical invasiveness and significantly improves fetal survival.

• Natural delivery enables pups to nurse from the dam, allowing extended postnatal observation.

• Use of green fluorescent protein (GFP)-expressing donor tissue permits real-time visualization of graft location and growth.

• The protocol is readily adaptable for xenotransplantation and studies of immunological tolerance during fetal development.

## Graphical overview


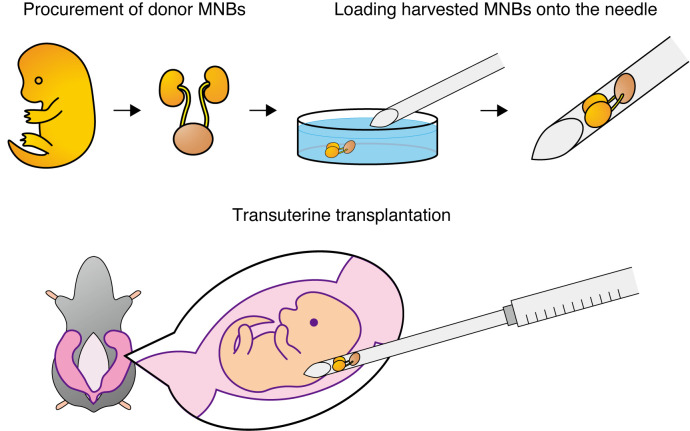


**Fetal kidneys with attached bladders are first harvested from donor fetuses.** Each fetal kidney–bladder unit is then loaded onto the bevel of a 15–16 G needle. After laparotomy in the pregnant rat, the uterus is exteriorized, and under a stereomicroscope, the needle is inserted transuterinely into the subcutaneous space of the recipient fetus. By expelling the contents of the needle, the donor fetal kidney is transplanted into the subcutaneous compartment of the fetus in utero. MNBs: metanephros–bladder units.

## Background

The fetal environment offers a niche rich in growth factors and immunologically permissive conditions, fundamentally different from that of adult tissues [1,2]. Capitalizing on this favorable fetal environment, our protocol enables whole-organ transplantation into rat fetuses with longitudinal monitoring of graft maturation, establishing advanced research on developmental organogenesis, fetal immunology, and regenerative medicine.

Previous in utero approaches have chiefly focused on microscale cell injections into the amniotic fluid, the peritoneal cavity, or the retroperitoneal space [3–6]. These methods accommodate fine-gauge needles, minimizing surgical trauma, but are limited to single-cell delivery and cannot support transplantation of intact organs. Whole-organ transplantation has remained challenging, as the use of larger-bore needles significantly increases fetal mortality. By introducing donor kidney–bladder units into the subcutaneous compartment, our protocol maintains fetal viability while providing a compliant space conducive to organ growth. This strategy overcomes the spatial limitation inherent to intraperitoneal and retroperitoneal delivery sites, enabling organ-scale transplantation during gestation. Furthermore, the transplanted donor kidneys become vascularized by recipient-derived vessels, allowing them to develop post-transplantation and form mature glomerular and tubular structures capable of producing urine.

Beyond modeling congenital kidney disease, this technique can be extended to evaluate xenogeneic grafts and investigate mechanisms of immune tolerance during gestation. It also offers potential for regenerative studies, as kidney organoids often exhibit limited maturation when implanted into adult hosts. Transplanting these organoids into fetal recipients may reveal developmental cues that promote more complete nephrogenesis. Therefore, this protocol not only fills a critical methodological gap in in utero transplantation but also opens new avenues for assessing organoid performance, immunological interventions, and organ-level developmental biology.

## Materials and reagents

**Biological materials**

1. Sprague–Dawley (SD) pregnant rats (Sankyo Labo Service Corp., Japan)

2. SD-Tg [CAG-enhanced green fluorescent protein (EGFP)] pregnant rats (also called GFP-SD rats) (Sankyo Labo Service Corp., Japan)

**Reagents**

1. Isoflurane (Pfizer, catalog number: 2817774)

2. Pentobarbital sodium (Kyoritsu Seiyaku Corp.)

3. Ethanol, 70% (for disinfection)

4. Hank’s balanced salt solution (HBSS) (Thermo, catalog number: 14025134)

**Laboratory supplies**

1. Surgical scissors: standard pattern with serrations (FST, catalog number: 14007-14)

2. Ophthalmic scissors: fine iris scissors (FST, catalog number: 14094-11)

3. Microforceps: Dumont medical micro-blunted atraumatic tipped forceps (FST, catalog number: 11253-25)

4. Ring forceps (Natsume Seisakusho Co., catalog number: A-26)

5. Castroviejo needle holder (FST, catalog number: 12565-14)

6. 5-0 silk suture (Natsume Seisakusho Co., catalog number: CF1250B2NT)

7. 15–16 G needle (Saito Medical Instruments Inc.)

8. 10 cm sterile culture dish (AS ONE Co., catalog number: GD90-15)

9. Sterile gauze

10. 1 mL syringe (TERUMO, catalog number: SS-01T)

11. Warming pad

12. Polystyrene box (size L260 mm × H175 mm × W160 mm)

13. Ice

## Equipment

1. Stereomicroscope (Leica Microsystems, model: M205FA)

2. Isoflurane anesthesia system (BioMedical Science, model: TK-40)

## Procedure

**A. Procurement of donor fetal kidneys with bladders**

*Note: Instead of transplanting a single fetal kidney, we employed an en bloc transplantation of the fetal kidney–ureter–bladder unit, termed the metanephros-bladder unit (MNB), to prevent hydronephrosis for a defined period [7,8]. The procedure for isolating the MNB is described below; see Video 1 for details.*

**Figure 1.**
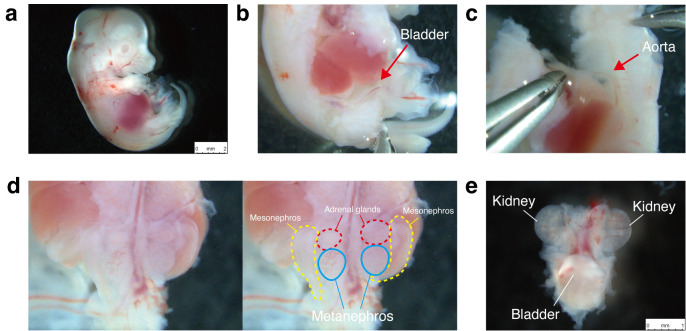
Isolation of the metanephros–bladder unit (MNB) from fetal Sprague–Dawley rats (E15.5). (a) Left lateral recumbent position of an E15.5 Sprague–Dawley fetus. (b) Identification of the bladder after thoracoabdominal incision. (c) Peeling the dorsal body wall, including the vertebral column, away from the trunk while ensuring the abdominal aorta remains attached to the trunk. (d) Bilateral mesonephroi and adrenal glands prior to excision. (e) Excised MNB.

1. Euthanize GFP-expressing pregnant Sprague–Dawley rats (E14.0–16.5) under isoflurane anesthesia by intravenous injection of pentobarbital (120 mg/kg body weight).

2. Incise the maternal skin and abdominal wall with surgical scissors, excise the entire uterus with fetuses intact, and transfer it to a 10 cm dish filled with HBSS.

3. Under a sterile stereomicroscope, open the myometrium and amnion with ophthalmic scissors, sever each umbilical cord, and remove the fetuses one by one.

4. After collecting all fetuses, place them in a fresh 10 cm dish containing HBSS and keep it on ice.

5. Under the stereomicroscope, isolate the metanephros–bladder units (MNBs) from each fetus.

6. Transfer a single fetus to a new 10 cm dish filled with HBSS. Working at room temperature with two microforceps, dissect the MNB within 10–15 min (practice is required to achieve this speed).

7. Decapitate the fetus, place it in the left lateral recumbent position (Figure 1a), and make a longitudinal incision in the right thoracoabdominal wall to expose the thoracic and abdominal cavities. Avoid damaging the visceral organs.

8. Extend the incision toward the umbilicus. Trace the umbilical vessels—running alongside the bladder toward the common iliac arteries to locate the bladder (Figure 1b).

9. Detach the connective tissue at the bladder neck and sever the rectum caudal to the bladder.

10. Rotate the fetus into the right lateral recumbent position.

11. As in step A7, make a longitudinal incision in the left thoracoabdominal wall and extend it toward the umbilicus.

12. Peel the back, including the vertebral column, away from the trunk, ensuring the abdominal aorta remains with the trunk to prevent detaching the kidneys. If the kidneys separate with the back, the bladder remains in the trunk, and the ureters will be severed (Figure 1c).

13. After peeling away the back, excise the bilateral mesonephroi and adrenal glands (Figure 1d).

14. Transect the abdominal aorta proximal to the kidneys along with the superior mesenteric artery and excise the kidneys and bladder en bloc.

15. Remove any extraneous tissue from the harvested MNBs, leaving only the kidneys and bladder.

16. Place the harvested MNBs (Figure 1e) in a fresh 10 cm dish containing HBSS on ice and keep them until transplantation. Perform transplantation within 1 h of harvest.

**Video 1.**
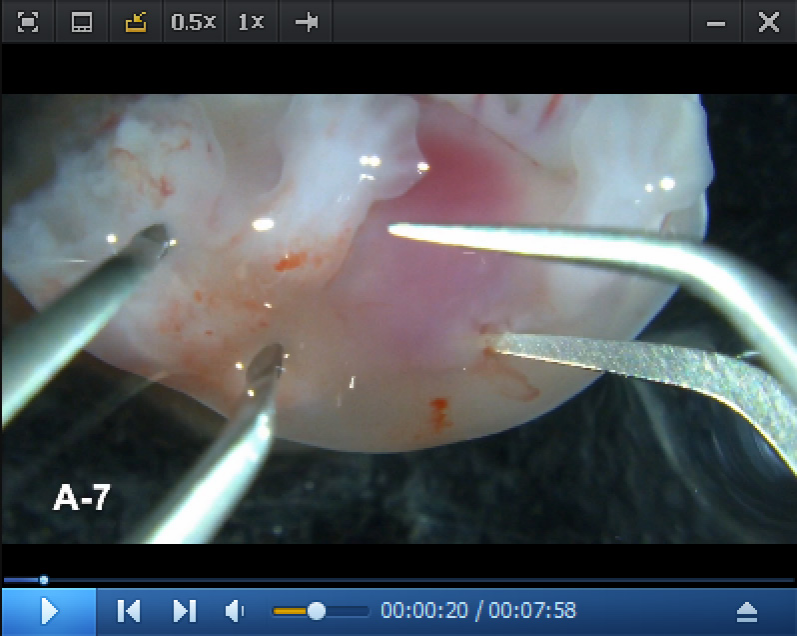
Dissection and retrieval of the metanephros–bladder unit (MNB) from Sprague–Dawley rat fetuses (E15.5)

**B. Loading the harvested MNBs onto the needle**

1. Attach a 1-mL syringe to a 15–16 G needle.

*Note: Use a custom thin-walled needle supplied by Saito Medical Instruments Inc., as a 15–16 G needle is the narrowest gauge that can accommodate the fetal kidney within the needle lumen.*

2. Fill a 10-cm dish nearly to the brim with HBSS to minimize air entry into the needle during loading.

3. Conduct all loading procedures under a stereomicroscope.

4. Transfer the harvested MNBs to the HBSS-filled dish prepared in step B2.

5. Fully submerge the bevel tip of the 15–16 G needle in HBSS and gently retract the plunger to fill the needle lumen, avoiding air bubbles. Approximately 0.1–0.2 mL of HBSS is sufficient. Keep the needle tip submerged throughout.

6. Under a stereoscopic view, bring the MNB and needle tip into the same field.

7. Hold the needle with the left hand and use microforceps in the right hand to gently guide the MNB—bladder end first—into the needle lumen.

8. Once both kidneys have entered the lumen, retract the plunger an additional 0.1 mL (approximately) so that the MNB is just inside the bevel. Avoid over-aspiration to prevent tissue damage (See Video 2 for steps B7–8).

9. After loading, remove the needle from the HBSS and place it horizontally in a sterile container.

10. To prevent desiccation, cover the needle tip with sterile gauze moistened with HBSS.

11. Keep the loaded needle at room temperature (22–27 °C); MNBs remain viable for up to 1 h post-loading.

**Video 2.**
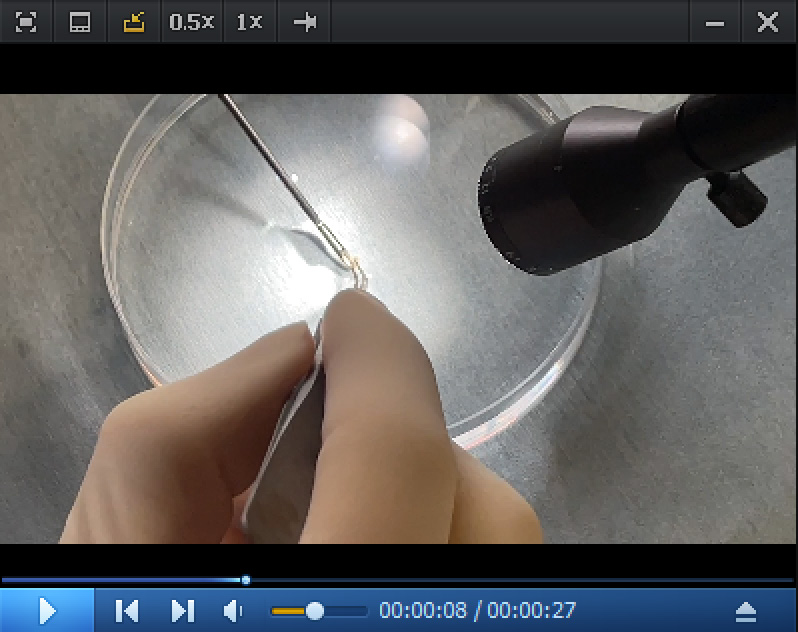
Loading the metanephros–bladder unit (MNB) onto the tip of a 15–16 G needle

**C. Transuterine transplantation**

**Figure 2.**
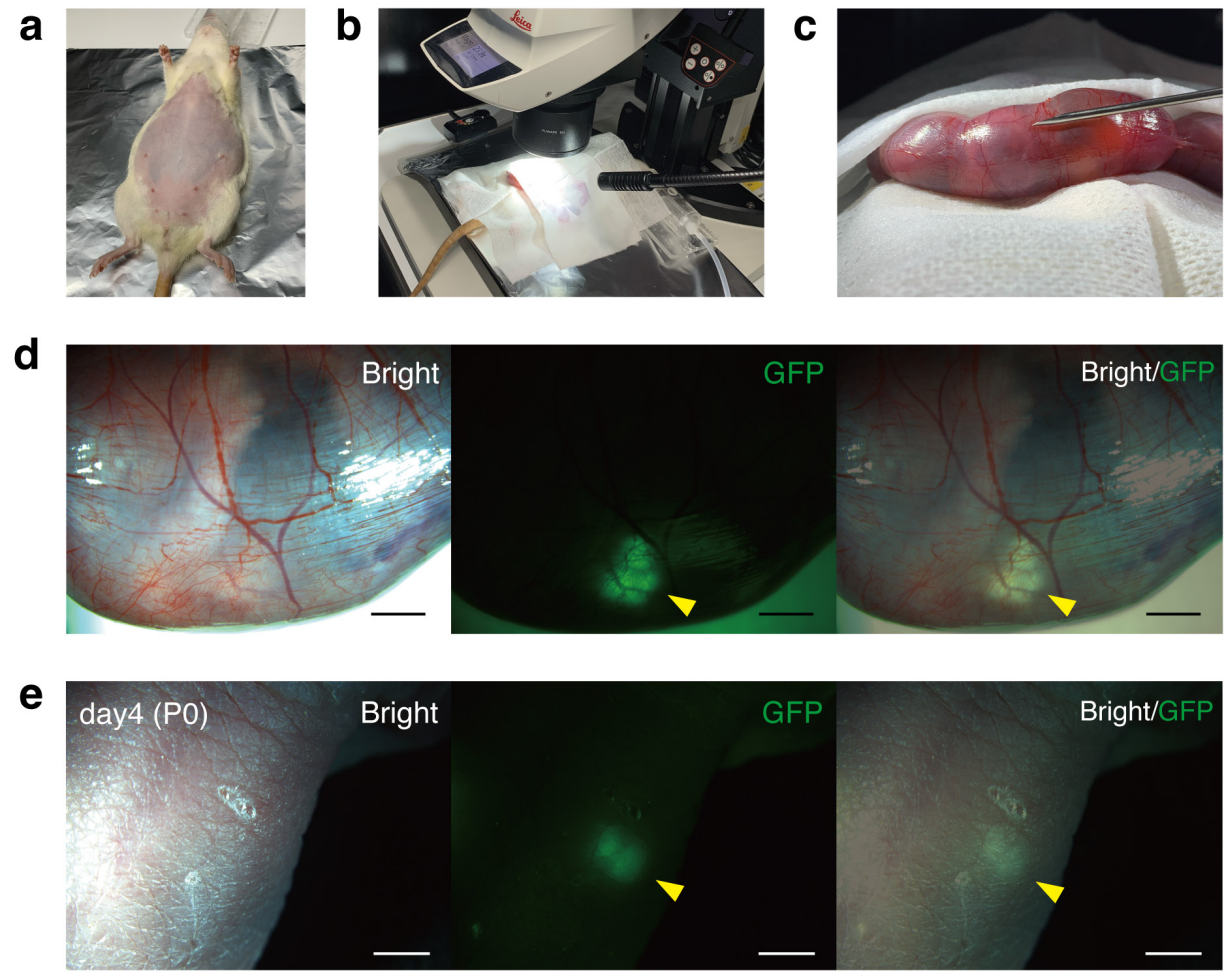
Transuterine transplantation of fetal metanephros–bladder units (MNBs) into recipient fetuses. (a) Pregnant Sprague–Dawley rat (E18.5) after abdominal shaving. (b) Entire uterus exteriorized and covered with sterile gauze moistened with 37 °C HBSS. (c) Thin uterine wall allows visualization of individual fetuses. (d) GFP-positive MNB visualized beneath the fetal skin immediately after transplantation. (e) GFP-positive transplanted MNB detected subcutaneously in the neonate after birth. Scale bars: 2 mm in (d) and (e). GFP, green fluorescent protein. Panels d and e are reproduced from Figure 1f and Figure 2c of [9], licensed under CC BY 4.0 (https://creativecommons.org/licenses/by/4.0/).

1. Perform all transplantations under a stereomicroscope; place a 37 °C warming pad beneath the stage.

2. Anesthetize pregnant Sprague–Dawley rats (E18.0–18.5) with isoflurane and position them supine on the warming pad.

3. Shave the abdomen, taking care not to injure the nipples (Figure 2a).

4. Disinfect the abdominal skin with 70% ethanol and drape with sterile gauze that has a central opening.

5. Make a midline laparotomy. Using ring forceps, gently exteriorize the uterus by grasping the uterine wall between fetuses; avoid contact with uterine vessels to prevent bleeding and disruption of fetal blood flow (Video 3).

6. Once the entire uterus is externalized, cover it with sterile gauze moistened with 37 °C HBSS to prevent drying. The thin uterine wall allows visualization of individual fetuses (Figure 2b,c).

7. Select recipient fetuses that are clearly visible, with the head oriented to the left of the dam and the tail to the right. Transplantation is still feasible, however, even when the fetal orientation is reversed (Figure 2c).

8. Hold the ring forceps in the left hand and the 15–16 G needle in the right hand and puncture.

9. Under stereoscopic vision, stabilize the target fetus and uterus with the ring forceps.

10. Pierce the cranial uterine wall and amniotic membrane with the needle.

11. Lower the needle almost parallel to the table and puncture the superficial lateral thoracic region of the fetus.

12. After skin penetration, advance the needle approximately 1 cm subcutaneously.

13. Release the ring forceps, grasp the needle with the left hand to immobilize it, and depress the plunger by 0.1 mL to expel the HBSS and the MNB (see Videos 4 and 5 for steps C8–13).

14. Observe a subcutaneous bulge filled with HBSS under the microscope.

15. Withdraw the needle and confirm GFP fluorescence to verify the correct placement of the GFP-positive MNB beneath the fetal skin. Proper subcutaneous placement causes minimal fetal bleeding; the uterine puncture hole is left unsutured (Figure 2d).

16. Repeat the procedure in 1–4 fetuses, limiting the total operative time (from anesthesia induction to closure) to ≤ 40 min; the exact timing depends on the operator’s skill. The duration of anesthesia is approximately 30 min.

17. Return the uterus to the abdominal cavity with ring forceps. Ensure no torsion, especially near the cervix, to avoid obstructing parturition.

18. Instill 5 mL of 37 °C HBSS into the peritoneal cavity to reduce postoperative adhesions.

19. Close the abdomen with 5–0 silk.

20. Discontinue anesthesia and return the dam to its home cage.

**Video 3.**
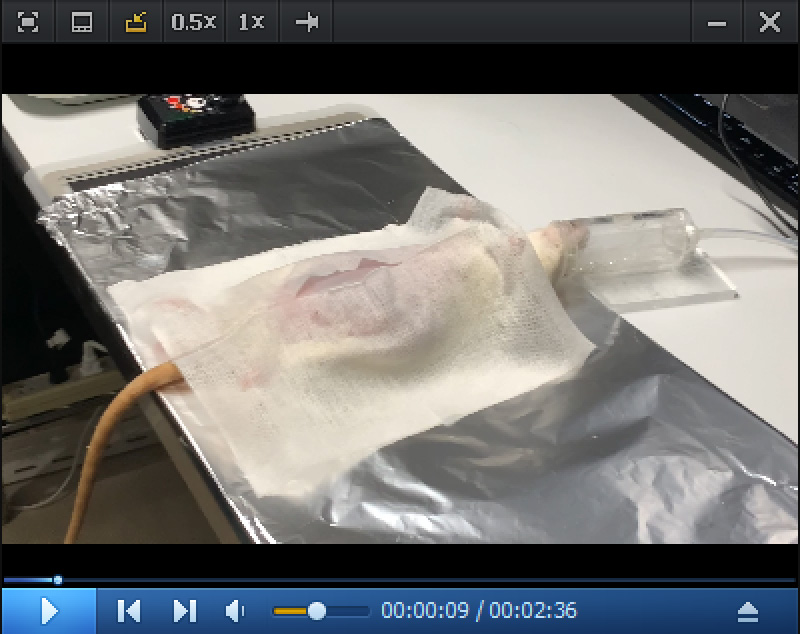
Gentle exteriorization of the uterus from pregnant Sprague–Dawley rats (E18.5)

**Video 4.**
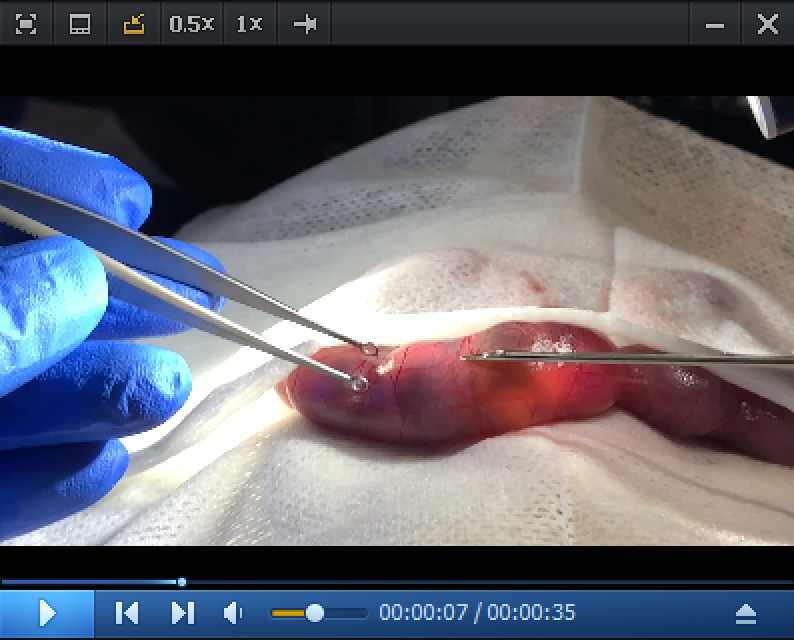
Transuterine transplantation of the metanephros–bladder unit (MNB) into the subcutaneous space of E18.5 fetal Sprague–Dawley rats using a 16 G needle (macroscopic view)

**Video 5.**
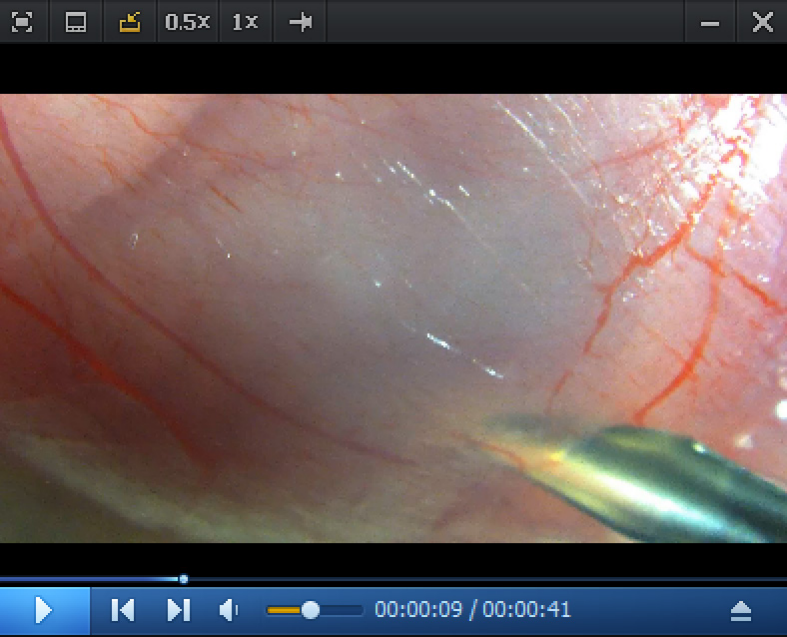
Microscopic view of transuterine transplantation of the metanephros–bladder unit (MNB) into the subcutaneous space of E18.5 fetal Sprague–Dawley rats using a 16 G needle

**D. Observation of the neonates**

1. Four days later (gestational day 22), allow natural delivery.

2. Briefly remove the pups from the cage and identify the transplanted individuals by GFP fluorescence under a stereomicroscope (Figure 2e).

## Validation of protocol

This protocol (or parts of it) has been used and validated in the following research article:

- Keita et al. [9]. Fetal-to-Fetal Kidney Transplantation in Utero *Communications Biology* (Figures 1–2 and Table 1). In the preceding study, this method was used to compare immune rejection and the degree of fetal kidney maturation between fetal-to-fetal transplantation and transplantation into adult recipients, using maturation markers such as nephrin (podocytes), LTL (proximal tubules), and E-cadherin (distal tubules).

For figures and videos relevant to this protocol, please refer to Figure 1 and Supplementary Video 1 in the cited reference [9].

The transplantation success rates shown in Table 1 of this protocol are reproduced from Table 1 of [9], licensed under CC BY 4.0 (https://creativecommons.org/licenses/by/4.0/).

**Table 1.** Transplant success rate [9] *The difficulty of the intrauterine transplantation procedure varies depending on the orientation and size of the fetus and the amount of amniotic fluid. In pregnant rat number 4, the transplantation technique was challenging, requiring extended time to transplant into a single fetus. To avoid prolonged anesthesia time, we completed the procedure after transplanting into just one fetus. In contrast, in pregnant rat number 2, the transplantation procedure was relatively straightforward, allowing us to successfully transplant into four fetuses.

Pregnant rat number	Number of live pups/all fetuses	Survival rate (%)	Number of transplanted fetuses	Number of GFP-positive neonates	Success rate (%)
1	12/14	86	2	1	50
2	9/11	82	4*	4	100
3	11/14	79	2	2	100
4	8/14	57	1*	1	100
Average	-	76	-	-	88

## General notes and troubleshooting

**General notes**

1. The procedure has succeeded only when Sprague–Dawley (SD) rats were used as recipients, whereas donor organs can be obtained from either rats or mice without difficulty.

2. Table 2 summarizes the temperature and timing conditions applied at each stage of the protocol.

**Table 2.** Temperature and timing conditions throughout the protocol

Phase	Material	Temperature	Duration	Related steps
Donor fetus collection	Pregnant SD rats (donor)	Room temperature (22–27 °C)	About 5 min	A1–3
Temporary storage of fetuses	Fetuses in HBSS (10 cm dish)	On ice	You may leave it for 1–2 h	A4
MNB dissection	Single fetus in HBSS (10 cm dish)	Room temperature (22–27 °C)	Complete within 10–15 min (per fetus)	A5–13
Storage of dissected MNBs	Harvested MNBs in HBSS	On ice	Transplant within 1 h after harvest	A14
Preparation for needle loading	HBSS in 10 cm dish	Room temperature (22–27 °C)	During loading procedure only	B4–8
Loaded needle storage	Needle containing MNBs	Room temperature (22–27 °C)	Use within 1 h after loading	B9–11
Recipient surgery–maternal temperature maintenance	Pregnant SD rats (recipient) placed on heating pad	37 °C	Entire surgery; total operative time ≤ 40 min	C1–20
Preventing uterine drying	Sterile gauze moistened with HBSS	HBSS warmed to 37 °C	While uterus is exteriorized	C6
Prevention of postoperative adhesions	HBSS (5 mL) instilled intraperitoneally	HBSS warmed to 37 °C	Immediately before abdominal closure	C18
Postoperative recovery	Dam after transplantation	Room temperature (22–27 °C)	From recovery to natural delivery (4 days)	C20, D1
Neonate identification	Pups during GFP screening	Room temperature (22–27 °C)	Short handling period only	

3. Because surgical fatigue can reduce transplantation success rates when multiple procedures are performed in a single session, we recommend limiting transplantation to one pregnant rat per day.

4. For the selection of recipient fetuses, those located near the cervical end of the bicornuate uterus are more likely to be delivered successfully than those positioned toward the ovarian end. Therefore, we recommend transplanting into fetuses closer to the cervix.

5. Successful transplantation requires that the dorsal side of the fetus be clearly visible under the microscope. Choose fetuses whose orientation makes the procedure easy (at a minimum, those with the back facing upward).

6. If the needle is inserted too deeply during subcutaneous puncture, bleeding may be observed from the fetus after needle withdrawal. Such fetuses have a high likelihood of dying; therefore, switch immediately to another fetus for transplantation.

7. If small air bubbles from inside the needle enter the fetal subcutaneous space during transplantation, they will dissipate spontaneously and do not cause problems.

8. When withdrawing the needle after expelling the fetal kidney, the graft may adhere to the needle tip and be pulled out of the fetus. Remove the needle carefully to avoid dislodging the graft.

9. The location of implantation (e.g., upper back vs. lower back) does not significantly affect postnatal graft development once GFP-positive tissue is confirmed after transplantation.

10. Monitor the pregnant dam daily after transplantation. If the transplanted fetus dies in utero, it may obstruct the birth canal at delivery, which can lead to maternal death.

## References

[1] Ander S. E., Diamond M. S. and Coyne C. B. (2019). Immune responses at the maternal-fetal interface. Sci Immunol. 4(31): eaat6114. https://doi.org/10.1126/sciimmunol.aat6114 PMC6744611 30635356

[2] Forbes K. and Westwood M. (2010). Maternal growth factor regulation of human placental development and fetal growth. J Endocrinol. 207(1): 1 16 16. 10.1677/joe-10-0174 20817666

[3] Shaw S. W., Peng S. Y., Liang C. C., Lin T. Y., Cheng P. J., Hsieh T. T., Chuang H. Y., De Coppi P. and David A. L. (2021). Prenatal transplantation of human amniotic fluid stem cell could improve clinical outcome of type III spinal muscular atrophy in mice. Sci Rep. 11(1): e1038/s41598–021–88559–z. 10.1038/s41598-021-88559-z PMC8080644 33911155

[4] Kihara Y., Tanaka Y., Ikeda M., Homma J., Takagi R., Ishigaki K., Yamanouchi K., Honda H., Nagata S., Yamato M., .(2022). In utero transplantation of myoblasts and adipose-derived mesenchymal stem cells to murine models of Duchenne muscular dystrophy does not lead to engraftment and frequently results in fetal death. Regen Ther. 21: 486 493 493. 10.1016/j.reth.2022.10.003 36313392 PMC9596598

[5] Flake A. W., Roncarolo M. G., Puck J. M., Almeida-Porada G., Evans M. I., Johnson M. P., Abella E. M., Harrison D. D. and Zanjani E. D. (1996). Treatment of X-Linked Severe Combined Immunodeficiency by in Utero Transplantation of Paternal Bone Marrow. N Engl J Med. 335(24): 1806 1810 1810. 10.1056/nejm199612123352404 8943162

[6] Yamanaka S., Saito Y., Fujimoto T., Takamura T., Tajiri S., Matsumoto K. and Yokoo T. (2019). Kidney Regeneration in Later-Stage Mouse Embryos via Transplanted Renal Progenitor Cells. J Am Soc Nephrol. 30(12): 2293 2305 2305. 10.1681/asn.2019020148 31548350 PMC6900792

[7] Yokote S., Matsunari H., Iwai S., Yamanaka S., Uchikura A., Fujimoto E., Matsumoto K., Nagashima H., Kobayashi E., Yokoo T., .(2015). Urine excretion strategy for stem cell-generated embryonic kidneys. Proc Natl Acad Sci USA. 112(42): 12980 12985 12985. 10.1073/pnas.1507803112 26392557 PMC4620909

[8] Kinoshita Y., Kobayashi E., Matsui K., Inage Y., Morimoto K., Yamamoto S., Iwai S., Kitada K., Iwasawa K., Saito Y., .(2025). Life-supporting functional kidney replacement by integration of embryonic metanephros-bladder composite tissue transplants. Kidney Int. 107(6): 1051 1063 1063. 10.1016/j.kint.2025.02.024 40122339 PMC12521919

[9] Morimoto K., Yamanaka S., Matsui K., Kinoshita Y., Inage Y., Yamamoto S., Koda N., Matsumoto N., Saito Y., Takamura T., .(2025). Fetal-to-fetal kidney transplantation in utero. Commun Biol. 8(1): e1038/s42003–025–07783–9. 10.1038/s42003-025-07783-9 PMC11876676 40033127

